# Bridging the gap: an economic case study of the impact and cost effectiveness of comprehensive healthcare intermediaries in rural Mexico

**DOI:** 10.1186/s12961-020-00563-3

**Published:** 2020-05-22

**Authors:** Anne Williamson, Lorena Ponce de León, Francisco Rodríguez Garza, Valeria Macías, Hugo Flores Navarro

**Affiliations:** 1 Compañeros en Salud/Partners in Health Mexico, Calle Primera Poniente Sur 25, Ángel Albino Corzo, 30370 Chiapas, Mexico; 2 Barts and the London School of Medicine and Dentistry, Queen Mary University of London, London, United Kingdom; 3 Bloomberg School of Public Health, Johns Hopkins University, Baltimore, MD United States of America; 4 Division of Global Health Equity, Brigham and Women’s Hospital, Boston, MA United States of America; 5 Harvard Medical School, Boston, MA United States of America

**Keywords:** Health systems, quality adjusted life years, cost-effectiveness analysis, health economics, rural health, Mexico

## Abstract

**Background:**

In rural settings where patients face significant structural barriers to accessing healthcare services, the formal existence of government-provided health coverage does not necessarily translate to meaningful care delivery. This paper analyses the effectiveness of an innovative approach to overcome these barriers, the Right to Health Care programme offered by Compañeros en Salud in Chiapas, Mexico. This programme provides comprehensive free coverage of all additional direct and indirect medical costs as well as accompaniment through the medical system. Over 550 patients had participated from 2013 until November 2018.

**Methods:**

Focusing on ten of the most frequently treated conditions, including hernias, cataracts and congenital heart defects, we performed a retrospective case study analysis of the quality-adjusted life years (QALYs) gained from treatment and the cost per QALY for 69 patients. This analysis used disability weights and uncertainty intervals from the Global Burden of Disease study and organisational micro-costing data for each patient. Each patient was compared to their own hypothetical counterfactual health outcome had they not received the secondary and tertiary care required for the specific condition. A mixed methods approach is used to establish this counterfactual baseline, drawing on pre-intervention observations, qualitative interviews and established literature precedent.

**Results:**

The programme was found to deliver an average of 14.4 additional QALYs (95% uncertainty interval 12.4–15.8) without time discounting. The mean cost per QALY over these conditions was $388 USD (95% UI $262–588) at purchasing power parity.

**Conclusions:**

These numbers compare favourably with studies of other health services and international cost per QALY guidelines. They reflect the on-treatment effect for the ten conditions analysed and are presented as a case study indicative of the promise of healthcare intermediaries rather than a definitive assessment of cost-effectiveness. Nonetheless, these results show the potential feasibility and cost effectiveness of a more comprehensive approach to healthcare provision in a resource-limited rural setting.

**Trial registration:**

This study involves economic analysis of a programme facilitating access to public healthcare services. Thus, there was no associated clinical trial to be registered.

## Key Messages

- Indirect medical expenses and geographic remoteness pose barriers to health service access even where universal health coverage is formally achieved.
- *Compañeros en Salud*, a non-governmental health organisation, implemented a Right to Health Care programme delivering an average of 14.4 additional quality-adjusted life years (QALYs) (95% uncertainty interval 12.4–15.8), at a mean cost per QALY of $388 USD (95% UI $262–588) at purchasing power parity for the cases analysed. These cases were patients treated by the programme for ten common diseases.
- These results suggest that the programme compares favourably to other interventions and cost-per-QALY guidelines and show the potential feasibility and cost-effectiveness of a comprehensive approach to health provision in resource-limited rural settings.

## Background

Formal provision of health services does not necessarily translate to meaningful access in the presence of poverty and other significant structural barriers [1]. These barriers include transport and accommodation costs, clinic and appointment wait times, geographic distances, additional medical expenses, and system complexity. While public healthcare services may cover direct medical costs, they rarely cover these indirect medical and non-medical expenses accruing to patients. These problems are particularly acute in rural areas, where unequal geographic distribution of doctors increases the challenge placed on individual patients [2].

Compañeros en Salud has implemented a system of free comprehensive health support under the Right to Health Care (Referencias) programme that is innovative in the extent of the medical and financial support provided. This operates in the Sierra Madre region of Chiapas, the poorest state in Mexico, where over 75% of the population lived in poverty in 2012 [3]. Since its inception in 2013, the programme has served over 550 patients, connecting rural primary care clinics to secondary and tertiary care facilities across Mexico by providing medical referrals, advice from doctors and accompaniment through the healthcare system, and financial support for medical and non-medical expenses, including transport, accommodation and food. These services fulfil a unique ‘intermediary’ function, because they provide the resources and information for patients to transition from a diagnosis in a primary care clinic to accessing the secondary and tertiary services provided by the Mexican Ministry of Health (Secretaría de Salud). Without additional medical advice and funding for services such as bus tickets to hospital, these patients would be unlikely to access the specialist care that was formally available to them. We aimed to determine whether comprehensive provision of this kind was cost-effective despite involving higher upfront costs, through facilitating improved system access and health outcomes.

The Right to Health Care programme is uniquely generous and comprehensive in its coverage of non-medical expenses amongst schemes identified in the literature. This is because it directly focuses on facilitating access to treatment and sets no upper limit on funding provision. No known scheme exists elsewhere in Mexico that is equivalent in terms of financial support or medical advice to patients. Analogous schemes exist in other countries to specifically fund travel for low-income individuals, including a United Kingdom National Health Service funding scheme for those receiving income support or other specified benefits [4] and a Medicaid coverage of non-emergency medical transportation in the United States [5]. A voluntary medical transport scheme in the English Midlands was found to improve the welfare of residents, though this study utilised geography tools without quantitative results, rather than health economic analysis [6]. However, these programmes do not extend to accommodation and food throughout the journey, nor do they include medical accompaniment by a doctor. To our knowledge, there are no analyses of the health impact or cost-effectiveness of any of these alternatives.

There is a range of relevant literature assessing health interventions in low-resource settings more broadly, including assessments of cataract and cleft lip surgeries, and a comparison of surgical programmes in Kenya and Canada [7–9]. Each of these utilised quality-adjusted life years (QALYs) or similar variables to quantify health improvements and several also captured cost-effectiveness in terms of cost per QALY. However, the current study fills a key gap in the literature by applying established analytical techniques to a unique type of healthcare scheme.

## Methods

### Setting

This health intervention took place in the Sierra Madre region of Chiapas, where Compañeros en Salud has operated since 2011. Previous health provision in this region was heterogeneous. Most communities have a medical station or clinic, but many of these have no stationed doctor or consistent medical supplies. Therefore, patients may face extremely long and expensive journeys to access care [10].

**Fig. 1:**
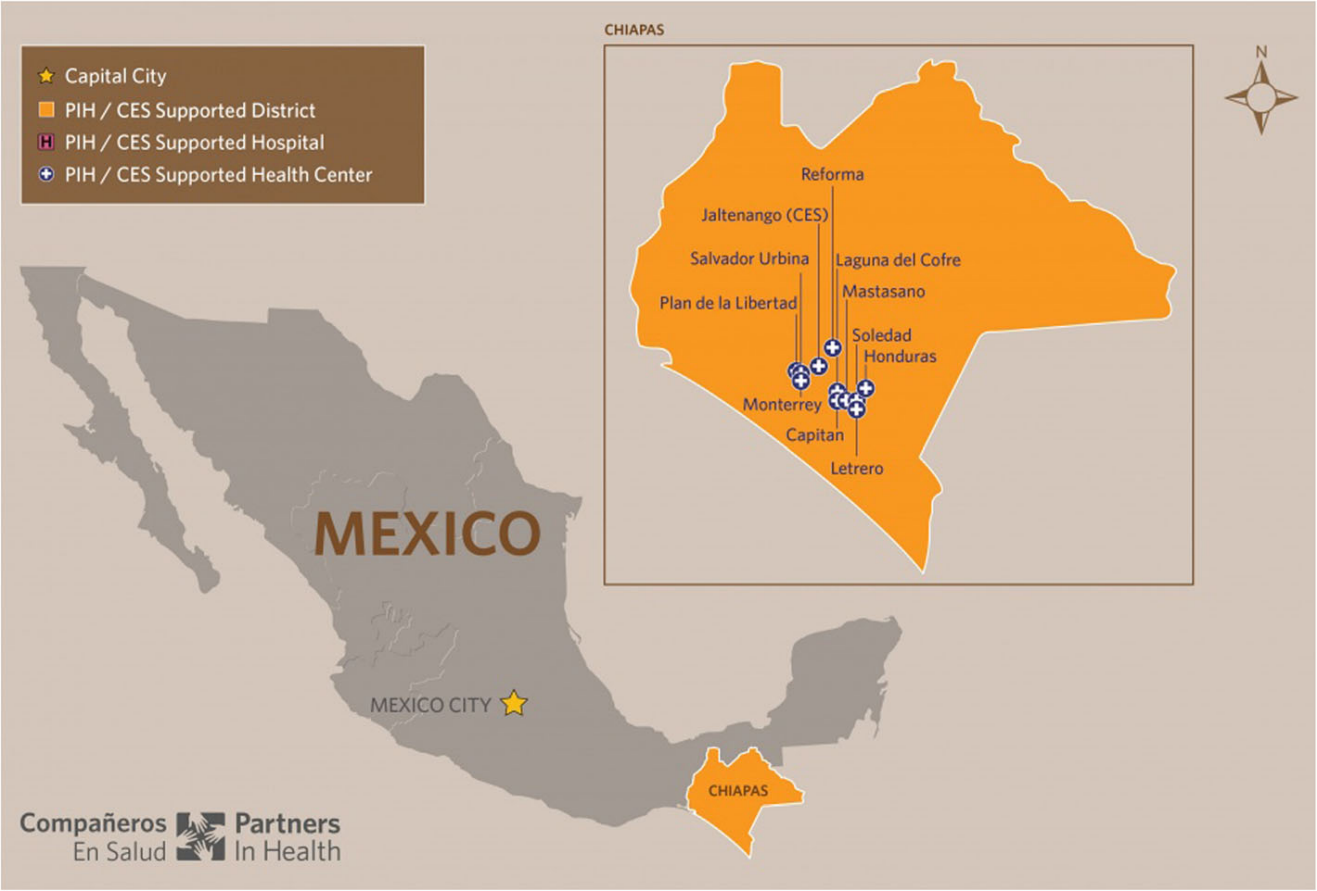
*Compañeros en Salud* locations

The intervention was created to enhance the pre-existing primary healthcare programme run by the organisation throughout this area, which consists of 11 rural medical clinics established and staffed in collaboration with the Secretariat of Health of Chiapas. These clinics provide primary care free at the point of delivery to a population of over 25,000 individuals, the vast majority of whom are covered by the government *Seguro Popular* health insurance programme (Fig. 1)

The Right to Health Care programme intervention begins with the identification of patients in need of more specialist medical care than can be provided at these primary clinics. They are referred to the most appropriate state secondary or tertiary services for initial consultation, largely located in the state capital, Tuxtla Gutiérrez, and then supported through the entire process of surgery, medication regimes or any other medical treatment required. A doctor or other appropriately trained staff member from the Right to Health Care programme accompanies patients to these consultations, providing additional support in navigating the medical system. All expenses are covered, including transport costs, accommodation as required to attend consultations, food during these travel periods, and any additional medical costs incurred.

All patients in communities served by these clinics must travel via Jaltenango de la Paz (Jaltenango), a major regional town with a population of approximately 10,000 people [11], where the organisation office is located. They continue on to Tuxtla Gutiérrez via a 3-hour journey on a mixture of paved and unpaved roads, and further travel to Mexico City or elsewhere proceeded onwards from there. A large proportion of patients live outside the specific communities where the organisation clinics are based and travel to these sites in order to receive care. This then entails further transportation time and expenses. The clinics vary in terms of their distance from urban centres, and therefore have been clustered in four groups for the purpose of this analysis. Those in the Jaltenango area, including the nearby towns of Reforma and Salvador Urbina, are closest to the organisation office in Jaltenango. This is followed by the semi-urban towns of Honduras and La Soledad, located close to one another in the Siltepec municipality with a combined population of over 1750, around 4-hours drive from Jaltenango via unpaved roads. Thirdly, Monterrey and Plan de La Libertad are a pair of small communities in La Concordia with a combined population of around 1000, also only accessible via difficult unpaved roads. Finally, Capitan, Letrero, Matasano and Laguna del Cofre are four extremely small communities in the Siltepec area, with all four together also totalling around 1000 people.

### Study design

We performed a cross-sectional, retrospective case study analysis of a subsample of the patient population (*n* = 550), presenting with a wide variety of conditions and treated under the Right to Health Care programme since its initiation in 2013. Some conditions were relatively common in this population such as cataracts (*n* = 13) or skin cancers (*n* = 11). However, many patients were the sole affected individual, or one of few patients, suffering from a particular illness. To preserve patient anonymity and increase the robustness of averaged results for specific conditions, this analysis focused on ten of the most frequently treated conditions in the programme: hernias, epilepsy, heart conditions, gall bladder conditions, cataracts, cryptorchidism, thyroid disorders, kidney stones, skin cancers and breast cancer. These conditions, and the average benefit to patients across these ten conditions, constitute the primary units of analysis.

A total of 67 patients completed treatment for one of the ten conditions selected, and thus were analysed in this study. Another 43 patients were diagnosed with one of the ten conditions selected but did not complete treatment through the programme. These patients were offered the same package of healthcare assistance and financial support as those who completed the programme. Nonetheless, there exist a range of reasons why they may have dropped out. Carefully considering each scenario, we decided to exclude these patients from this analysis, and therefore our results reflect the on-treatment effect rather than an intention to treat effect. The key reasons for dropping out were, firstly, patients who individually decided not to pursue an invasive surgery or treatment with side effects. Secondly, some patients decided to utilise other health services for the same condition, for instance, Guatemalan patients who chose to cross back to Guatemala for treatment in their home country. Because patients in both cases did not receive treatment from the Right to Health Care programme following initial enrolment, neither costs nor benefits could be attributed and these patients were excluded. Further, some patients were lost to follow-up after their initial appointments, ceasing to attend before an assessment of any health improvement could be made. These patients were also excluded because of a lack of sufficient information to determine any health improvements.

Finally, a further 13 patients were part way through treatment for one of the selected conditions when this analysis was conducted. Because the health benefits to these patients could not be accurately determined, they were also excluded from the analysis. Two patients in the programme died of their primary disease or another condition during the early stages of enrolment in the programme, before completing treatment. They were included in the analysis as having gained zero QALYs, because we felt unable to retrospectively estimate any health improvements during their life that could be attributed to the programme. In order to avoid division by zero in calculating the cost per QALY for these patients, their costs were set to nil and were not included in cost per QALY averages across the sample.

**Fig. 2:**
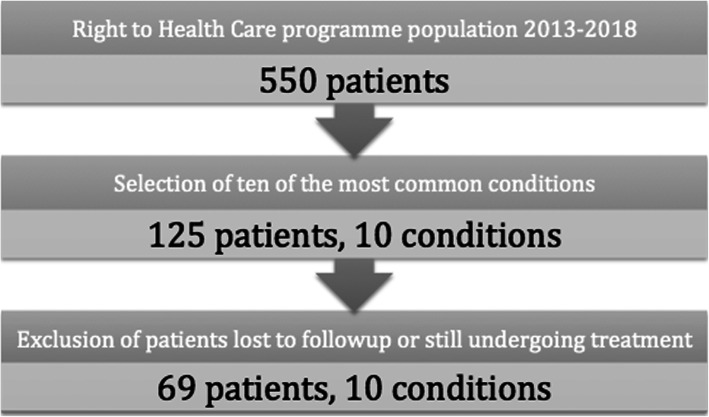
Patient sample selection process

These steps resulted in a final sample size of 69 patients. The steps involved are presented in Fig. 2. This sample selection is discussed under Descriptive Statistics in the Results section, where a statistical comparison of patients included and excluded from the Right to Health Care programme is presented. We conclude there that there was no systematic demographic variation between the two groups that was likely to bias results. However, because complete information on the health status and later trajectory of excluded patients was unavailable, we could not rule out bias arising if these patients would have responded differently to treatment to those who were analysed. Thus, we qualify our results and present them as a case study of patients who completed treatment for the ten conditions analysed.

We reviewed organisational records on all patients treated under the Right to Health Care programme for one of the ten conditions in question. This involved drawing on detailed spending data for each patient as well as utilising health records from the organisation’s primary care clinics to determine health status before and after treatment.

To assess the impacts of the programme, we compared each patient treated to their own hypothetical counterfactual health outcome had they not received the secondary and tertiary care required for the specific condition. This baseline was based on prior observations of patients in local primary care clinics, finding that patients did not access full advanced care before the implementation of the Right to Health Care programme. In making the assumption that patients’ conditions would not have improved otherwise, we followed established precedent in the literature analysing cost effectiveness of health interventions in low- and middle-income settings [7–9]. The validity of this baseline assumption is considered in the Discussion. We also conducted qualitative interviews with a subsample of patients analysed (*n* = 17), to inform our choice of baseline for this case study. These interviews were conducted orally. They asked patients whether they had experienced financial stress due to their medical condition, and whether financial barriers had prevented them from receiving medical care prior to enrolment in the Right to Health Care programme.

### Study outputs

We consider two primary analytical outputs: the health improvement achieved by each patient, measured in terms of QALYs gained, and the cost per QALY gained. One QALY is equivalent to a full year of perfect health, and each year lived with a given medical condition is allocated a proportion of this full value based on revealed preference surveys capturing the disutility of the condition. The QALY measures patient health by combining patient life expectancy with a disability weighting for any health conditions, and thus captures both mortality and morbidity components of health states.

We used disability weights from the Global Burden of Disease (GBD) Study 2016 [12], the most recent year available, to assign a health-related quality of life weight for each year of a patient’s life where the GBD study covered the condition in question. These weights represent the magnitude of health loss associated with specific health outcomes and are widely used to quantify the effectiveness of health interventions. The GBD study estimates the burden of diseases for 195 countries, and the associated weights have been utilised for a wide range of Mexico-specific research [13, 14]. For conditions not covered by the GBD study, namely cryptorchidism and skin cancer, disability weights were taken from established literature focusing on Latin America where possible. We also use the uncertainty intervals in the GBD study as a form of probabilistic sensitivity analysis for our conclusions. The weights used for each condition are included in Appendix 1.

The quality of life weight attached to a given year of life without specific treatment or surgery was defined as *Q* = 1 − *D*, following Sassi [15], where *D* was the disability weight attached to the condition. These weights range from 0, which equals full health, to 1, which equals death. The actual or expected health-related quality of life weight for each year after treatment was similarly defined as *Q*^*i*^ = 1 − *D*^*i*^, where *D*^*i*^ was the disability weight attached to the post-treatment health state.

QALYs gained were calculated using the following formula, adapted from Sassi [15] to explicitly separate the recovery and post-recovery periods:

$$ QALYs\ gained=\sum \limits_{t=a}^{a+R-1}\frac{Q_t^R}{{\left(1+r\right)}^{t-a}}+\sum \limits_{t=R+a}^{a+{L}^i-1}\frac{Q_t^i}{{\left(1+r\right)}^{t-a}}-\sum \limits_{t=a}^{a+L-1}\frac{Q_t}{{\left(1+r\right)}^{t-a}} $$

This formula calculates the quality-adjusted life expectancy during the post-intervention recovery period and following this recovery, and subtracting the quality-adjusted life expectancy without treatment from the sum of these two. Here, *L* is the residual life expectancy in years of the individual at age *a* without treatment, *R* is the number of years of recovery required to reach full post-intervention levels of functioning, and *t* represents individual years within that life expectancy range. *L*^*i*^ refers to the residual life expectancy of the individual following treatment, ${Q}_t^i$ is the health-related quality of life weight attached to each year of life following treatment and recovery, and ${Q}_t^R$ is the health-related quality of life weight in the recovery period. Finally, *r* is the time discount rate. A minor alteration from Sassi [15] is that time is calculated from the end of each discrete year, so *L = 0* on the day of the patient’s death rather than when they have a year to live. Calculation of QALYs gained was conducted in Microsoft Excel, and descriptive statistics were analysed using IC version 15 (StataCorp, College Station, TX). We calculated QALYs gained rather than disability-adjusted life years averted in order to focus on the beneficial impact of this programme for patients’ health instead of framing its effect as ‘minimising disability’. Although in some contexts the two measures may deviate, we argue that *Q* = 1 − *D* provides a good approximation of a quality-adjusted life year for this indicative case study. Further analysis would be enhanced by direct elicitation of quality of life weights from the community where the health intervention has taken place.

Life expectancies were calculated using nation level data for Mexico scaled down by 98.2% and 96.8% for men and women, respectively, to obtain a life expectancy at each age for inhabitants of Chiapas – a state with life expectancies substantially lower than other parts of Mexico. The scaling factor was derived by dividing the predicted 2016 life expectancy at birth in Chiapas, 72.7 years for men and 76.7 years for women [12], by the overall Mexican life expectancy at birth; 74 for men and 79.2 for women [16]. Life expectancies without intervention were derived using established life table practices and disease-specific mortality statistics from the existing literature. These life tables are included in Appendices 2 and 3.

Analysis of QALYs gained was conducted twice; once with a time discount rate of 3% as is standard in much of the economic literature, and once with a discount rate of 0%. We argue in favour of 0% time discounting as costs are also not discounted, as we outline below, and consistent discounting for costs and outcomes would be appropriate [17]. Nonetheless, these results are presented in parallel given the wide prevalence of a 3% time discount factor in the cost-effectiveness literature.

Costs were then calculated using patient treatment records, as detailed data are collected on consultations, travel plans and expenses paid for by the authors’ organisation. These spending data divide into four categories. First, transport costs were covered for the patient and an accompanying family member, such as bus tickets from Jaltenango to Tuxtla Gutiérrez costing 200 Mexican pesos ($22 USD) each for a return journey, and further bus expenses for journeys to other secondary or tertiary hospitals in nearby cities Tapachula, Revolución, Villaflores or, occasionally, Mexico City. Patient petrol expenses were also covered where they organised personal travel to consultations. Second, accommodation and food during travel to and from appointments was paid for, with a voucher of 25 Mexican pesos ($2.66 USD) given per traveller per meal, and specific hostels organised to host patients in each of the cities where hospitals were located. Third, any additional charges for medical or surgical consultations were covered by the organisation, of which detailed records were kept using the CommCare software. Fourth, any additional laboratory tests or studies required by specialists were paid for, again with detailed records kept. This detailed microdata at the patient level means that overall average programme costs can be accurately calculated, and used to determine overall cost effectiveness.

We also calculated an average value of organisational capital and overhead costs, taking data on the office rental, petrol for operational use, maintenance and upkeep of rooms in the office that were provided for patients’ accommodation and wages, and apportioning 20% of general organisational costs and 75% of Right to Health Care staff wages to the programme. These figures were chosen because the Right to Health Care programme was one of the five roughly equally sized components of organisational operations, thus warranting 20% of the overall costs. The programme utilised 75% of the monthly time of designated staff, who also allocated some focus to the development of a separate surgical programme. This resulted in monthly costs of 68,134 pesos, or $7263 USD, calculated at 2018 purchasing power parity (PPP) [18]. This total cost was divided by the average number of monthly appointments in 2018, namely 110, to arrive at a value per appointment of 619 pesos or $66 USD. This cost was added for each patient appointment, including those that were missed by the patient or the doctor.

All of these costs were taken in 2018 terms. This means that even patients treated prior to 2018, whose costs would have been slightly lower in nominal terms due to inflation, were considered as though they were treated at the same time as 2018 patients. This allowed us to overcome missing data on transport and accommodation prices in previous years, and meant that no discount factor was applied to costings as the analysis treated all costs as incurred during the same period.

## Results

### Descriptive statistics

The demographics of the analysed patients were compared to all other patients from the Right to Health Care programme patient population at large to examine the internal validity of findings for the programme as a whole. These results are presented in Table 1.

**Table 1:** Comparison of demographics between analysed and excluded Right to Healthcare programme patients

	Analysed		Excluded		Total
Respondents	*n*	%	*n*	%	
All	69		481		550
Gender					
Male	33	48	199	41	232
Female	36	52	282	59	318
Age					
0–18	20	29	121	25	141
19–64	26	38	264	55	290
65+	23	33	51	11	74
Unknown	0	0	39	8	39
Clinic					
Jaltenango area (Jaltenango, Reforma and Salvador Urbina)	28	41	157	33	185
Honduras and Soledad	10	14	94	20	104
Capitan, Letrero, Matasano and Laguna del Cofre	14	20	154	32	168
Monterrey and Plan de la Libertad	17	25	76	16	93

Percentages may not sum to 100% due to rounding

χ^2^ squared independence tests were conducted for each variable and found that there was no significant gender bias or bias in clinic cluster location at the 5% level when comparing the analysed sample relative to the overall patient population. However, there was a significant relationship between age and inclusion in the analysed group, with more patients analysed being either under 18 or over 65 years. In particular, a greater proportion of those analysed were over 65, 33% compared to 11% of the overall population. If anything, this would lead to an underestimation of the benefits of the programme, because a younger patient population will, all things being equal, gain more QALYs as a result of an intervention because they have more years expected to live. Overall, we conclude the sample was broadly representative and not significantly biased.

### Health impacts and cost-effectiveness

The programme was estimated to deliver an average of 14.8 (95% uncertainty interval (UI) 12.8–16.2) additional QALYs without time discounting, or 6.5 (95% UI 5.3–7.4) additional QALYs with 3% annual time discounting. The mean cost per QALY over these conditions was $388 USD PPP (95% UI $262–588) without time discounting, or $494 (95% UI $339–747) with 3% discounting. These results are presented by condition in Table 2.

**Table 2:** Primary outcomes, overall and by condition (95% uncertainty interval)

Condition	# Treated	Mean QALYs gained		Mean cost per QALY (USD, PPP)	
Discount factor		0%	3%	0%	3%
All	69	14.4 (12.4–15.8)	6.3 (5.2–7.2)	383 (262–588)	488 (339–747)
Skin cancer	11	3.8 (2.5–5.3)	3.0 (2.0–4.1)	513 (371–781)	583 (421–889)
Cataract	13	2.0 (1.3–2.8)	1.2 (0.8–1.8)	1284 (809–1995)	1506 (949–2339)
Severe	5	4.4 (3.1–6.3)	2.6 (1.8–3.7)	591 (420–850)	713 (507–1025)
Moderate	8	0.4 (0.3–0.7)	0.3 (0.2–0.6)	1717 (1051–2711)	2001 (1225–3160)
Hernia	6	30.3 (28.9–32.1)	9.9 (8.9–11.1)	31 (27–36)	63 (55–73)
Severe	2	15.7 (14.0–17.9)	9.6 (8.3–11.2)	66 (54–80)	86 (69–107)
Moderate	4	37.7 (36.4–39.2)	10.0 (9.3–11.0)	14 (13–14)	52 (47–56)
Heart conditions	6	26.7 (26.1–27.4)	9.1 (8.7–9.7)	51 (44–61)	137 (116–165)
Severe	4	29.6 (29.1–30.1)	10.1 (9.6–10.7)	40 (39–40)	113 (108–119)
Moderate	2	20.8 (20.2–21.9)	7.1 (6.8–7.7)	67 (50–93)	173 (129–234)
Epilepsy	8	24.5 (21.3–26.9)	8.9 (6.9–10.4)	44 (41–53)	103 (90–134)
Severe	5	20.0 (16.8–21.4)	8.8 (6.8–10.0)	63 (58–77)	137 (121–180)
Less severe	3	32.0 (28.8–36.0)	9.0 (7.2–11.2)	13 (12–14)	46 (37–58)
Gall bladder conditions	4	26.2 (23.5–29.2)	11.2 (9.5–13.2)	17 (16–19)	41 (35–47)
Cholecystitis	3	29.2 (26.0–32.7)	12.5 (10.5–14.9)	15 (14–17)	36 (30–43)
Cholelithiasis	1	17.2 (16.2–18.4)	7.3 (6.6–8.1)	23 (21–25)	54 (49–60)
Thyroid conditions	4	7.0 (4.5–9.4)	3.8 (2.5–5.2)	56 (42–88)	100 (74–157)
Kidney stones	6	4.0 (2.7–5.6)	2.1 (1.4–2.9)	304 (218–444)	568 (407–830)
Breast cancer	5	29.9 (29.8–30.0)	18.4 (18.3–18.6)	77 (77–78)	124 (123–124)
Cryptorchidism	6	13.6 (5.4–13.6)	6.0 (2.4–6.0)	57 (57–144)	129 (129–324)

In general, results varied across treated conditions, with the greatest number of QALYs gained for patients with moderate hernias and severe heart conditions. Cataracts were associated with the lowest number of QALYs gained, followed by skin cancers. In part, this is explained by the fact that these conditions also had the oldest average age of patients treated, and thus health improvements, even if significant, were not expected to affect as many future years of life. However, all these values are strong in comparison to international benchmark figures. For instance, the United Kingdom’s National Institute for Health and Care Excellence suggest a ‘threshold’ of £20,000 to £30,000 per QALY, equivalent to $28,571 to $42,857 USD [19]. Further comparisons are outlined in the Discussion.

### Sensitivity analysis

A second dimension of sensitivity analysis considers alternative counterfactuals to the core assumption that all patients would not have received treatment in the absence of the Right to Health Care programme. We test the sensitivity of the results to this assumption by instead assuming that 10% of patients would have received the full care they required under Seguro Popular if they did not have access to the Right to Health Care programme. This, by mathematical construction, results in a 10% decrease in estimated average QALYs gained to 13.0 QALYs (95% UI 11.2–14.2) and an increase in average cost per QALY of slightly more than 10% to $426 (95% UI $291–653). It could be further tested assuming a range of possible scenarios, such as 15% or 20% of patients obtaining care under a counterfactual where this intervention did not provide for them.

With any of these three alternative assumptions, the results are still extremely positive, and the costs involved are orders of magnitude below international threshold guidelines under both scenarios. Further, we argue that this more cautious counterfactual is less likely to accurately reflect this specific patient population under consideration than the core assumption of no alternative treatment. This is due to the impoverished and extremely rural setting of the intervention, the information gaps which present significant hurdles to obtaining the correct medical care without support, and credit constraints including high interest rates or complete lack of access to credit. These factors combine to mean that if patients did not have access to the Right to Health Care programme, the vast majority would experience these medical costs as catastrophic expenditure. Although some patients would doubtless seek to obtain treatment independently without such a programme, we strongly argue that very few, if any, would successfully be able to complete the full course of treatment necessary to obtain the required health benefits. This assumption is further considered in the Discussion section. Nonetheless, we recognise that the assumption of a 100% treatment effect is a very stringent one, and that it would not necessarily hold in all settings outside of this case study. Thus, these results should be taken as indicative of one end of the spectrum of potential benefits of an expansive healthcare access programme. It is likely that similar obstacles to healthcare access may apply to certain other populations in poor rural areas of both low- and middle-income countries, but the counterfactual assumption should be adjusted to a level appropriate for the specific setting.

## Discussion

Universal health coverage has long been a key aim in the global health landscape, notably included in the WHO Sustainable Development Goals under Target 3.8: “*Achieve universal health coverage, including financial risk protection, access to quality essential health-care services and access to safe, effective, quality and affordable essential medicines and vaccines for all*” [20].

In many low- and middle-income settings, much of the population cannot access full health services when sick in spite of this goal being officially achieved. Mexico presents a paradigm case of this challenge, although the Constitution of Mexico also enshrines the right to health protection to all citizens [21]. The Mexican government established the Seguro Popular public health insurance programme in 2002 in an effort to cover those previously without insurance [22]. As a result, the proportion of the population without any health insurance shrunk from 51.62% in 2006 to approximately 25% in 2012, equating to over 50 million additional citizens with health coverage [23]. However, the country continues to be plagued by marked regional inequalities in access and quality, with particular health differences between urban and rural areas and across socio-economic groups [2].

To our knowledge, the Right to Health Care intervention is the first to focus specifically on bridging the gap between existing public health systems and patients in need. At the time of this intervention, Seguro Popular was the flagship nationwide financial scheme aimed at reducing healthcare costs for patients using a pre-defined package of services (it has since been replaced by a similar programme called Insabi). However, the structural barriers to access mean that the delivering organisation fulfils a critical role in allowing these to be overcome. This may be one of the reasons why an intermediary scheme is so cost-effective; it is filling in the gaps in the system in order to unlock the benefits and services already formally on offer.

There were two channels through which this programme fulfilled its intermediary function; first, financial support for all individuals required direct and indirect costs, and second, the provision of doctors to accompany patients through the medical system. The individual effects of each of these two channels were not distinguished, but we argue that the barriers of additional expenses and a confusing system exacerbate one another. For instance, bureaucratic complexity led to surgery cancellations for several of our patients, which simultaneously increased indirect costs and would also likely deter patients without professional advice. We suggest that the combination of these two channels is required to make formal universal coverage into a reality in the rural Mexican setting. Thus, one policy lesson is that a streamlined system that is more easily comprehended by patients also presents lower barriers to access.

It should be noted that the Right to Health Care programme did not cover the entirety of any patient’s health expenses. It is integrated into the primary healthcare services provided by this organisation through the clinics, with patients referred to participate in the programme when doctors or other health providers identified a need for more advanced care than could be provided in this rural setting. This interconnected approach is what allows for holistic care, follow-up and such good health outcomes. The government also covers some medical costs for specific conditions under Seguro Popular, although many patients within this system were required to cover certain medical expenses themselves, such as laboratory tests or the cost of surgical equipment – for example, hernia mesh – due to funding limitations in the public system. Nevertheless, the full health improvement attained by our patients as a result of their treatment was attributed to the programme. We consider this approach valid based on a key inference – these health improvements were not attained before involvement of Compañeros en Salud.

The patients supported were, in general, previously isolated and cut off from the public system that exists to provide for them. Therefore, this programme is credited with the benefits attained just as, for example, a home visit health programme is evaluated for cost-effectiveness without including the government costs of the GP surgeries that patients are referred to, or the cost of constructing the roads required to access patients. This study focused on the marginal gain in QALYs obtained as a result of additional support from the Right to Health Care programme, relative to what would have occurred in its absence.

Is this a reasonable inference? We must compare outcomes to the most plausible counterfactual but establishing clear baseline data regarding health outcomes without the intervention is a major challenge for this study and for similar analyses in rural and resource-limited settings. Although Mexico maintains excellent health data, this is unable to shed light on those who fail to present to hospitals in the first place due to the kinds of structural barriers raised in this study and it is these non-attenders who are required for a perfect counterfactual.

For this case study, we instead developed a picture of likely counterfactual outcomes for these patients if the programme in question did not exist. We first draw on observations of patients in local primary care clinics, finding that patients did not access full advanced care before the implementation of the Right to Health Care programme. To further validate this statement, we conducted interviews with 17 of the patients in our cohort. These numbers are too small to constitute a representative sample of Right to Health Care programme patients, but provide an indication of whether it is reasonable to assume, as we do, that those treated here would not have received the required secondary or tertiary care without the authors’ organisation. Of the 17 interviewed, 14 reported that they had previously been advised to seek specialist care for the condition in question but missed these appointments due to financial barriers. Sixteen claimed to have experienced significant financial stress as a result of medical expenses before this organisation began providing free support. One patient explained; “*I was extremely worried about how to repay my previous medical debts. It was always on my mind, and I would talk to people about it – how am I going to get by?*” We argue that the specific context of local health provision, combined with the aforementioned literature precedent for using zero health improvement as the baseline in limited resource settings [7–9], makes such an approach valid here. However, the same inference may not hold in otherwise comparable settings where structural barriers to healthcare exist, such as rural areas in high-income countries where loans, personal savings or other avenues may facilitate access to secondary and tertiary medical care even without an intermediary provider like the Right to Health Care programme.

The QALYs gained by patients in this programme, on average and within each condition analysed, are substantial. This represents meaningful health improvements, reduced mortality and morbidity, as well as significant wider benefits to the families and communities of those treated. These results provide clear evidence of the health benefits from providing an intermediary between established health services and the most disadvantaged target users of the system in the Chiapas context. Given that QALYs gained are the basis of this study, the skew towards older patients may negatively bias the results as equivalent health improvements deliver more additional QALYs to a younger patient demographic. Thus, the programme may in fact result in more QALYs gained on average across the patient population than is found in this sample. However, we argue that the fact that the analysed sample also includes a higher proportion of those under 18 than the overall patient population mitigates this bias. The conditions analysed also indicate the epidemiological transition that Mexico is currently undergoing, with a shift towards a larger share of older adults in the population. Thus, the skew towards older patients in the data may in fact make these results more durable than otherwise, as the population moves towards the age distribution of the analysed sample.

Just as impressive are the cost per QALY figures. Any well-designed health system places patient outcomes as the foremost priority, over and above ‘cost savings’. However, appropriate cost-effectiveness analysis can facilitate this goal rather than working in opposition to it. Ensuring that interventions are cost-effective allows a limited pool of resources to extend further, and deliver more health improvements, and thus should be an important element in decision-making [24].

Given the centrality of cost-effectiveness considerations, a scheme like the Right to Health Care programme may be initially viewed as overly generous in its coverage, extending far beyond even the most comprehensive public health programmes worldwide. It is therefore particularly noteworthy that the health benefits derived from this additional support are so great that the average cost per QALY gained is only $388 USD (PPP). These numbers compare favourably with studies of other health services treating the same conditions, with a literature survey identifying international programmes covering similar conditions with a cost per QALY ranging from $696 USD per QALY for hernias to $2020 USD per QALY for cataracts [25–28]. Moreover, the financial benefits for the patients and their families are even more significant than these results suggest. The cost of obtaining this care independently would be greater for patients than that incurred by a centralised charity, as the majority do not have their own vehicles or know how to navigate the medical system efficiently, and would also likely have to accrue loans with significant associated interest if they were even able to pursue the requisite care.

These numbers also lie well below a number of international cost per QALY guidelines. A ratio of $50,000 per QALY has long been used as a benchmark for cost effectiveness in the United States [29], whilst as referenced in the Results section, the United Kingdom’s National Institute for Health and Care Excellence set a ‘threshold’ for recommending NHS treatments equivalent to $28,571 to $42,857 USD per QALY [19]. Other decision-makers previously recommended assessment relative to national income, such as the WHO’s suggestion of a cost-effectiveness threshold of 1 to 3 times per capita GDP [30]. In the case of Mexico, in 2017, this would give a range from $18,258 to $54,774 USD (PPP) per QALY [31]. Woods et al. [32] utilize a different method accounting for opportunity costs of spending, and arrive at a range of $422 to $1967 for El Salvador, broadly comparable to Mexico in terms of poverty and geographic location. The choice of QALY threshold is a contentious one, because it implies limiting patient access to certain beneficial treatments and also highlights extreme inequalities in global healthcare. However, in the case of this study, it is particularly notable that the Right to Health Care programme costs less than each of these potential thresholds, and lies below the upper limit of the given ranges for all conditions analysed.

## Conclusions

The Right to Health Care programme was found to deliver a significant health benefit for the analysed population and also to be cost-effective by a range of metrics. We posit that a similar intermediary programme would be of significant value to rural and vulnerable individuals across Mexico and many other low- and middle-income countries, and would likely be cost-effective. We also suggest that government or charitable providers use their scale to negotiate discounted arrangements for these services in the way Compañeros en Salud was able to. Based on insights gained in a rural Mexican setting, health policy planners should consider providing more expansive coverage for medical and non-medical expenses in rural areas as this would increase access to the existing system, improving health outcomes in an economically viable way. Having invested huge sums of money in establishing secondary and tertiary level health services, an effective programme facilitating healthcare access is essential to deliver the transformative benefits of good health.

## Data Availability

The datasets generated and/or analysed during the current study are not publicly available due to confidentiality requirements. Selected data are available from the corresponding author on reasonable request.

## Appendix 1

**Table 3:** Disease-specific disability weightings, IHME Global Burden of Disease Study 2016 [33] unless otherwise stated

Condition	Disability weight
Skin cancer	
Diagnosis and primary therapy phase of skin melanoma or other neoplasms^a^	0.29 (0.19–0.40)
Moderate disfigurement due to squamous cell carcinoma	0.07 (0.04–0.10)
Disfigurement due to basal cell carcinoma	0.01 (0.01–0.02)
Hernia	
Severe	0.32 (0.22–0.44)
Moderate	0.11 (0.08–0.16)
Cataract	
Severe vision loss	0.18 (0.13–0.26)
Moderate vision loss	0.03 (0.02–0.05)
Heart conditions	
Severe congenital heart disease and heart failure	0.21 (0.15–0.29)
Moderate congenital heart disease and moderate heart failure	0.07 (0.05–0.10)
Mild congenital heart disease and mild heart failure	0.04 (0.03–0.06)
Epilepsy	
Severe epilepsy, > 1 seizure per month	0.55 (0.38–0.71)
Less severe epilepsy, 1–11 seizures per year	0.26 (0.17–0.37)
Gall bladder conditions	
Cholecystitis: severe symptomatic gallbladder disease	0.32 (0.22–0.44)
Cholelithiasis: moderate symptomatic gallbladder disease	0.11 (0.08–0.16)
Hypothyroidism or hyperthyroidism	
Moderate endocrine, metabolic, blood and immune disorders	0.15 (0.10–0.20)
Kidney stones/nephritis	0.11 (0.08–0.16)
Breast cancer	
Primary diagnosis and therapy phase	0.29 (0.19–0.40)
Post-mastectomy	0.08 (0.05–0.12)
Cryptorchidism	
Classified as other congenital birth defect	0.22 (0.08–0.22)

^a^Similar to diagnosis disability weight of 0.26 applied for skin cancer by Soerjomataram et al. [34]

^b^Same disability weight as used in Poenaru et al. [9]

## Appendix 2

**Table 4:** Healthy life expectancy table, WHO [16] scaled to obtain Chiapas values

Age	Chiapas life expectancy (rounded to nearest year)	
Gender	Male	Female
0–1 year	73	77
1–4 years	74	78
5–9 years	74	78
10–14 years	74	78
15–19 years	74	78
20–24 years	75	79
25–29 years	75	79
30–34 years	76	79
35–39 years	76	80
40–44 years	77	80
45–49 years	77	80
50–54 years	78	81
55–59 years	79	82
60–64 years	81	83
65–69 years	82	84
70–74 years	84	86
75–79 years	86	87
80–84 years	89	90
85–89 years	91	93

## Appendix 3

**Table 5:** Additional mortality rate, by condition

Condition and explanation	Additional mortality (%), annual unless specified
Skin cancer	
No additional mortality rate assumed	–
Hernia	
4.5% probability of strangulation after 2 years of age [35] multiplied by a 50% mortality rate if strangulation is not treated within 48 h [36], given rural setting and lack of ambulance services	2.25
Cataract	
No additional mortality rate assumed	–
Heart conditions	
Atrial septal defect mortality rate per decade used to construct life expectancy table for untreated condition [37]	0.6–7.5
Aortic stenosis mortality rate 26% annually [38]	26
Moderate heart failure 1-year mortality rate of 24.5% in South American meta-analysis [39]	24.5
Epilepsy	
Mortality rate from uncontrolled epilepsy in rural Bolivia 0.97% annually, with similar mortality rates in other rural Latin American settings [40, 41]	0.97
Gall bladder conditions	
2.017% annual additional rate, from 36.3% disease-specific cumulative mortality after 18 years [42]	2.017
Hypothyroidism or hyperthyroidism	
No additional mortality rate assumed	–
Kidney stones	
No additional mortality rate assumed	–
Breast cancer	
Untreated median survival of 2.3 years [43]	2.3 years
Cryptorchidism	
No additional mortality rate assumed	–

## Abbreviations

PPP: purchasing power parity
QALYs: quality-adjusted life years
UI: uncertainty interval

## References

[1] Ensor T, Cooper S (2004). Overcoming Barriers to Health Service Access and Influencing the Demand Side Through Purchasing.

[2] OECD (2016). OECD Review of Health Systems: Mexico 2016.

[3] CONEVAL, Medición de la Pobreza: Resumen Ejecutivo. 2013. https://www.coneval.org.mx/Informes/Coordinacion/Pobreza_2012/RESUMEN_EJECUTIVO_MEDICION_POBREZA_2012_Parte1.pdf. Accessed 5 May 2020.

[4] NHS. Healthcare Travel Costs Scheme (HTCS). 2017. https://www.nhs.uk/using-the-nhs/help-with-health-costs/healthcare-travel-costs-scheme-htcs/. Accessed 12 Mar 2020.

[5] Chaiyachati KH, Moore K, Adelberg M (2018). Too early to cut transportation benefits from medicaid enrollees. Health Services Insights.

[6] Sherwood KB, Lewis GJ (2000). Accessing health care in a rural area: an evaluation of a voluntary medical transport scheme in the English Midlands. Health Place.

[7] Lansingh VC, Carter MJ, Martens M (2007). Global cost-effectiveness of cataract surgery. Ophthalmology.

[8] Poenaru D (2013). Getting the job done: analysis of the impact and effectiveness of the SmileTrain Program in alleviating the global burden of cleft disease. World J Surg.

[9] Poenaru D, Pemberton J, Frankfurter C, Cameron BH (2015). Quantifying the disability from congenital anomalies averted through pediatric surgery: a cross-sectional comparison of a pediatric surgical unit in Kenya and Canada. World J Surg.

[10] Secretaría de Salud (2009). Unidades De Primer Nivel De Atencion En Los Servicios Estatales De Salud. Evaluación 2008.

[11] PueblosAmerica (2018). Jaltenango De La Paz.

[12] IHME. Mexico - Chiapas: How long do people live? 2016. http://www.healthdata.org/mexico-chiapas. Accessed 12 Mar 2020.

[13] Stevens G, Dias RH, Thomas KJA (2008). Characterizing the epidemiological transition in Mexico: national and subnational burden of diseases, injuries, and risk factors. PLoS Med.

[14] Gómez-Dantés H, Fullman N, Lamadrid-Figueroa H (2016). Dissonant health transition in the states of Mexico, 1990–2013: a systematic analysis for the Global Burden of Disease Study 2013. Lancet.

[15] Sassi F (2006). Calculating QALYs, comparing QALY and DALY calculations. Health Policy Plan.

[16] World Health Organization (2016). Life tables by country - Mexico.

[17] Parkinson B, Lourenço RDA. Discounting in Economic Evaluations in Health Care: A Brief Review. Sydney: Cancer Research Economics Support Team: University of Technology; 2015.

[18] OECD. Purchasing Power Parities (PPP). 2018. https://data.oecd.org/conversion/purchasing-power-parities-ppp.htm. Accsessed 11 Mar 2020.

[19] Dillon SA. Carrying NICE Over the Threshold | Blog | News. NICE. 2015.https://www.nice.org.uk/news/blog/carrying-nice-over-the-threshold. Accessed 12 Mar 2020.

[20] United Nations General Assembly. Transforming our World: The 2030 Agenda for Sustainable Development. A/RES/70/01. 2015. http://www.un.org/ga/search/view_doc.asp?symbol=A/RES/70/1&Lang=E. Accessed 1 Mar 2020.

[21] Constitución Política de los Estados Unidos Mexicanos - Ordenamiento - Legislación. 2018. https://www.juridicas.unam.mx/legislacion/ordenamiento/constitucion-politica-de-los-estados-unidos-mexicanos. Accessed 2 July 2019.

[22] World Bank (2015). Seguro Popular: Health Coverage For All in Mexico.

[23] Urquieta-Salomón JE, Villarreal HJ (2016). Evolution of health coverage in Mexico: evidence of progress and challenges in the Mexican health system. Health Policy Plan.

[24] Woodhall M (2004). Cost-benefit analysis in educational planning.

[25] Heaney DC, Shorvon SD, Sander JWAS (1998). An economic appraisal of carbamazepine, lamotrigine, phenytoin and valproate as initial treatment in adults with newly diagnosed epilepsy. Epilepsia.

[26] Busbee BG, Brown MM, Brown GC, Sharma S (2002). Incremental cost-effectiveness of initial cataract surgery. Ophthalmology..

[27] Stylopoulos N, Gazelle GS, Rattner DW (2003). A cost-utility analysis of treatment options for inguinal hernia in 1,513,008 adult patients. Surg Endosc.

[28] Johner A, Raymakers A, Wiseman SM (2013). Cost utility of early versus delayed laparoscopic cholecystectomy for acute cholecystitis. Surg Endosc.

[29] Neumann PJ, Cohen JT, Weinstein MC (2014). Updating cost-effectiveness — the curious resilience of the $50,000-per-QALY threshold. N Engl J Med.

[30] Bertram Melanie Y, Lauer Jeremy A, De Joncheere Kees, Edejer Tessa, Hutubessy Raymond, Kieny Marie-Paule, Hill Suzanne R (2016). Cost–effectiveness thresholds: pros and cons. Bulletin of the World Health Organization.

[31] World Bank (2017). World Development Indicators.

[32] Woods B, Revill P, Sculpher M, Claxton K (2016). Country-level cost-effectiveness thresholds: initial estimates and the need for further research. Value Health.

[33] Global Burden of Disease Collaborative Network. Global Burden of Disease Study 2016 (GBD 2016) Disability Weights. Seattle: Institute for Health Metrics and Evaluation; 2017.

[34] Soerjomataram I, Lortet-Tieulent J, Ferlay J (2012). Estimating and validating disability-adjusted life years at the global level: a methodological framework for cancer. BMC Med Res Methodol.

[35] Gallegos NC, Dawson J, Jarvis M, Hobsley M (1991). Risk of strangulation in groin hernias. Br J Surg.

[36] Abbas MH (2005). Outcome of strangulated inguinal hernia. Pakistan J Med Sci.

[37] Campbell M (1970). Natural history of atrial septal defect. Heart.

[38] Chizner MA, Pearle DL, deLeon AC (1980). The natural history of aortic stenosis in adults. Am Heart J.

[39] Ciapponi A, Bardach A, Calderón M (2015). Burden of heart failure in Latin America: a systematic review and meta-analysis. Value Health.

[40] Nicoletti A, Sofia V, Vitale G (2009). Natural history and mortality of chronic epilepsy in an untreated population of rural Bolivia: a follow-up after 10 years. Epilepsia..

[41] Levira F, Thurman DJ, Sander JW (2017). Premature mortality of epilepsy in low- and middle-income countries: a systematic review from the Mortality Task Force of the International League Against Epilepsy. Epilepsia.

[42] Ruhl CE, Everhart JE (2011). Gallstone disease is associated with increased mortality in the United States. Gastroenterology.

[43] Johnstone PAS, Norton MS, Riffenburgh RH (2000). Survival of patients with untreated breast cancer. J Surg Oncol.

